# Supplementary material for: Risk factors associated with sexually transmitted infections and HIV among adolescents in a reference clinic in Madrid
Source: PLoS One. 2020 Mar 16;15(3):e0228998. doi: 10.1371/journal.pone.0228998 (PMC7075699; doi:10.1371/journal.pone.0228998)
Supplement: S1 File — (PDF) [file pone.0228998.s001.pdf]

ADOLESCENTES, ITS Y VIH EN MADRID

Introducción: Los adolescentes tienen mayor incidencia de infecciones de transmisión sexual (ITS) que las personas con edades más avanzadas. La OMS, destaca la necesidad de adoptar programas integrales de prevención específicos dirigidos a este grupo etario. El objetivo de este trabajo fue analizar la prevalencia de las ITS/VIH entre los adolescentes e identificar los marcadores sociodemográficos, clínicos y conductuales asociados a estas infecciones, con el fin de promover estrategias preventivas específicas.

Metodología: Estudio descriptivo retrospectivo de los adolescentes, de 10 -19 años, atendidos en primera consulta entre 2016-2018 en una clínica monográfica de ITS de Madrid. A todos los adolescentes de les pasó un cuestionario epidemiológico estructurado donde se recogió información sobre características sociodemográficas, clínicas y conductuales. En función del riesgo individual, se efectuó un cribado de VIH, VHA, VHB, VHC, sífilis, gonococia y clamidiasis. El procesamiento y análisis de los datos se realizó mediante el paquete estadístico STATA 15.0

Resultados: La frecuencia de las ITS detectadas entre todos los adolescentes fue: gonococia 21,7%, clamidiasis 17,1%, sífilis 4,8% y VIH 2,4%. Tras realizar un análisis multivariante, las variables que de manera independiente y estadísticamente significativa se relacionaron con la presencia de alguna ITS fueron: la edad temprana de la 1ª relación sexual y tener antecedentes de ITS. El origen latinoamericano rozó el nivel de significación estadística (p=0.066).

Discusión/Conclusiones: Los adolescentes que comienzan de forma precoz las relaciones sexuales o los que tienen antecedentes de ITS presentan mayor riesgo para adquirir ITS. Se deben implementar programas preventivos combinados dirigidos específicamente a los adolescentes más jóvenes.
